# Supplementary material for: Liver group 2 innate lymphoid cells regulate blood glucose levels through IL-13 signaling and suppression of gluconeogenesis
Source: Nat Commun. 2022 Sep 15;13:5408. doi: 10.1038/s41467-022-33171-6 (PMC9478157; doi:10.1038/s41467-022-33171-6)
Supplement: Supplementary file 1 — Supplementary Information [file 41467_2022_33171_MOESM1_ESM.pdf]

Supplementary Information

Liver group 2 innate lymphoid cells regulate blood glucose levels through IL-13 signaling and suppression of gluconeogenesis

Masanori Fujimoto[1,2], Masataka Yokoyama[1], Masahiro Kiuchi[3], Hiroyuki Hosokawa[4], Akitoshi Nakayama[1], Naoko Hashimoto[1], Ikki Sakuma[1], Hidekazu Nagano[1], Kazuyuki Yamagata[1], Fujimi Kudo[5], Ichiro Manabe[5], Eunyoung Lee[6], Ryo Hatano[6], Atsushi Onodera[3, 7], Kiyoshi Hirahara[3], Koutaro Yokote[2], Takashi Miki[6, 8], Toshinori Nakayama[3, 9], Tomoaki Tanaka[2, 8*]

1 Department of Molecular Diagnosis, Graduate School of Medicine, Chiba University, Chiba, Japan.
2 Department of Endocrinology, Hematology and Gerontology, Graduate School of Medicine, Chiba University, Chiba, Japan.
3 Department of Immunology, Graduate School of Medicine, Chiba University, Chiba, Japan.
4 Department of Immunology, Tokai University School of Medicine, Isehara, Kanagawa, Japan.
5 Department of Systems Medicine, Graduate School of Medicine, Chiba University, Chiba, Japan.
6 Department of Medical Physiology, Chiba University, Graduate School of Medicine, Chiba, Japan.
7 Institute for Advanced Academic Research, Chiba University, Chiba, Japan.
8 Research Institute of Disaster Medicine, Chiba University, Chiba, Japan.
9 AMED-CREST, AMED, Otemachi, Tokyo, Japan.

*Address for correspondence
Corresponding Author: Tomoaki Tanaka (email: tomoaki@restaff.chiba-u.jp)
1-8-1 Inohana, Chuoku, Chiba, Japan

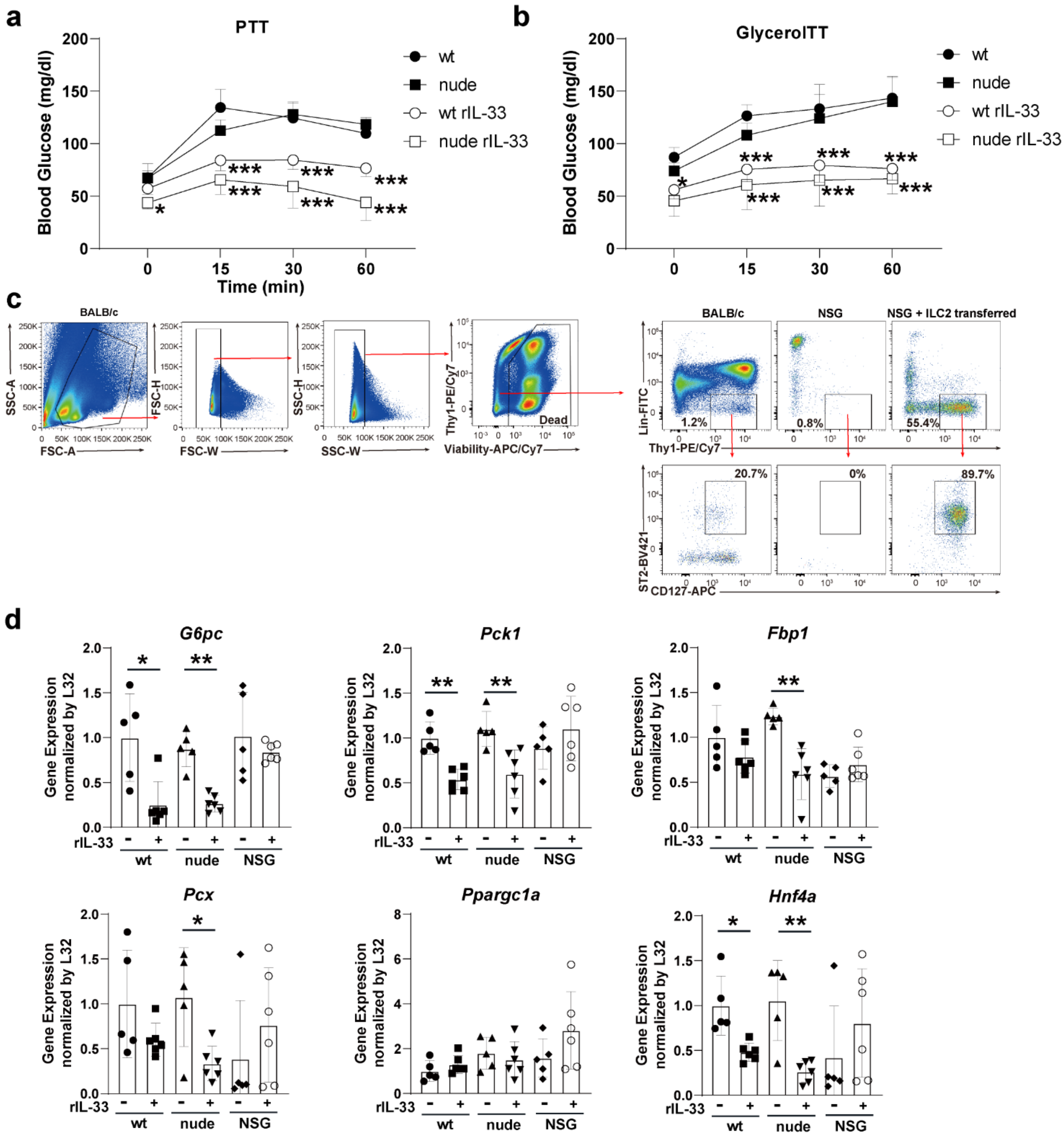

Supplementary Figure 1. | Recombinant IL-33 (IL-33) injection exerts a blood-glucose-lowering effect by limiting gluconeogenesis, and liver group 2 innate lymphoid cells (ILC2) may mediate this effect. Phosphate-buffered saline (PBS) or rIL-33 was injected for 5 consecutive days into wild-type (wt) or nude mice. After overnight fasting, blood glucose levels were measured in each mouse before and after intraperitoneal injection of pyruvate or glycerol. **a,** Blood glucose levels in wt BALB/c mice and nude mice as measured by the pyruvate tolerance test (2 g/kg of body weight, wt: n=7, wt rIL-33: n=5, nude: n=6, nude rIL-33: n=4). **b**, Blood glucose levels in wt and nude mice as measured

by the glycerol tolerance test (2 g/kg of body weight, wt: n=5, wt rIL-33: n=5, nude: n=6, nude rIL-33: n=4). **c**, Representative plot of gating for $Lin^{-}Thy1^{+}CD127^{+}ST2^{+}$ ILC2s from wt BALB/c, nontransferred NOD/Scid/$Il2R\gamma^{null}$ (NSG) and liver ILC2-transferred NSG mice. **d**, Real-time quantitative PCR (RT–qPCR) analysis of gluconeogenesis-related genes using liver tissues. PBS or rIL-33 was injected for 5 consecutive days into wt, nude or NSG mice (wt rIL-33[-]: n=5, wt rIL-33[+]: n=6, nude rIL-33[-]: n=5 nude rIL-33[+]: n=6 NSG rIL-33[-]: n=5, NSG rIL-33[+]: n=6). Unpaired one-sided Student's t test. $^{*}P < 0.05$; $^{**}P < 0.01$; $^{***}P < 0.001$. Each bar and its error bars represent the mean ± SD.

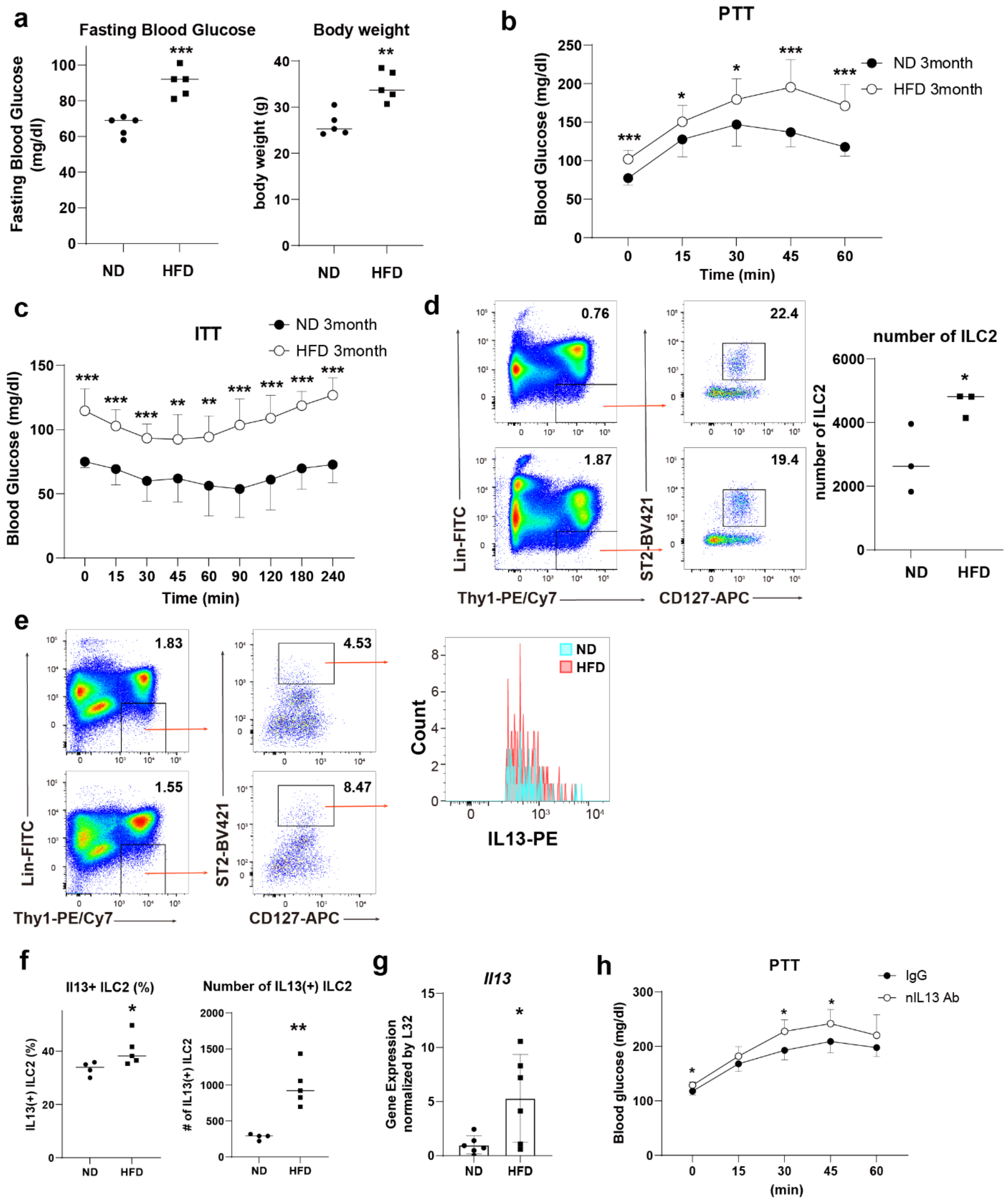

Supplementary Figure 2. | Three-month high-fat diet (HFD)-fed mice show excessive gluconeogenesis, which is exacerbated by IL-13 neutralization. a, Fasting blood glucose levels and body weights of 3-month normal diet (ND)-fed mice or HFD-fed mice (n = 5 per group). **b**, Blood glucose levels as measured by the pyruvate tolerance test (PTT; 2 g/kg of body weight, ND: n=8, HFD: n=9). **c,** Blood glucose levels as measured by the insulin tolerance test (0.1 U/kg body weight insulin, ND: n=8, HFD: n=9). **d,** Gating for Lin^{-}Thy1^{+}CD127^{+}ST2^{+} group 2 innate lymphoid cells (ILC2) and

the number of liver ILC2s in 3-month ND- or HFD-fed mice (left). Numbers of liver ILC2s (n=3 per group, right). **e,** Gating for Lin$^-$Thy1$^+$CD127$^+$ST2$^+$ ILC2s (left) and intracellular IL-13 in 3-month ND- or HFD-fed mice. **f,** Lin-Thy1+CD127+ST2+ ILC2s in liver tissues were evaluated. Percentages of IL-13(+) ILC2s in total ILC2s (left) and numbers of IL13(+) ILC2s (right). **g,** RT–qPCR analysis of *Il13* in liver tissues from ND- or HFD-fed mice (n=6 per group). **h**, Mice were fed a HFD for 3 months and intraperitoneally injected with control IgG or a neutralizing IL-13 antibody for 4 consecutive days. Then, blood glucose levels were measured by the PTT (2 g/kg of body weight, n = 4 per group). Unpaired one-sided Student's *t* test. $^*P < 0.05$; $^{**}P < 0.01$; $^{***}P < 0.001$. Each bar and its error bars represent the mean ± SD.

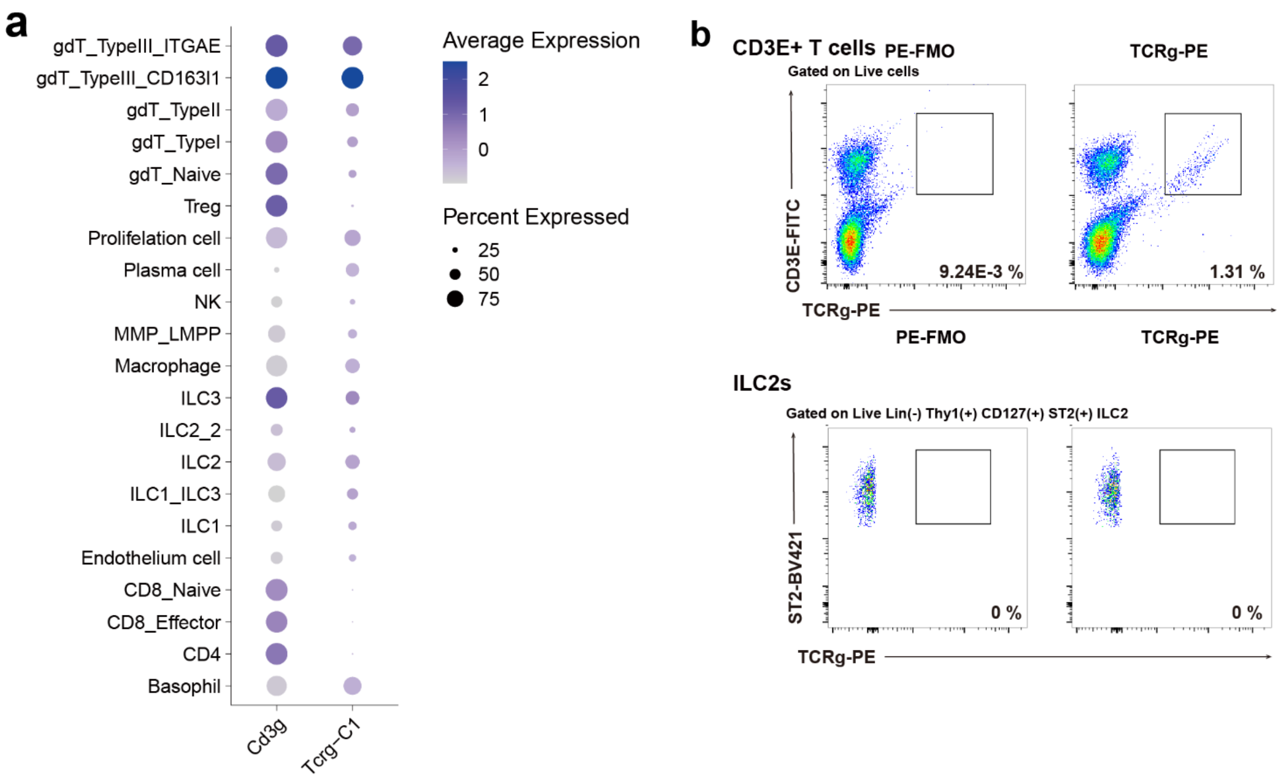

Supplementary Figure 3. | *Cd3g* and *Tcrg-C1* are barely expressed on group 2 innate lymphoid cells (ILC2) compared with other cell types. a, Dot plot showing the gene expression of *Cd3g* and *Tcrg-C1* in all types of cells. **b,** Flow cytometry analysis of TCRg-PE in CD3E+ T cells or ILC2s from liver tissue.

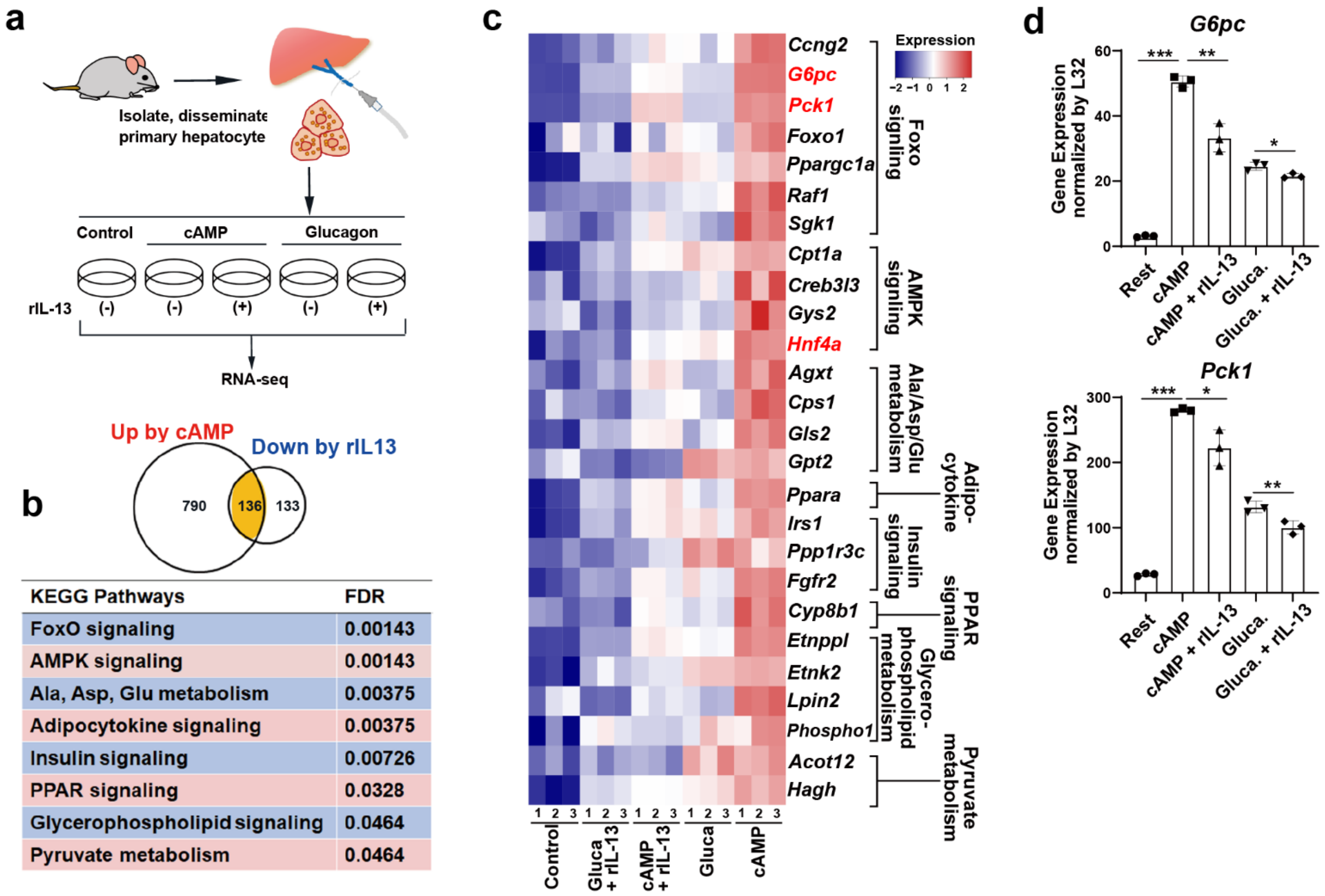

Supplementary Figure 4. | Recombinant IL-13 (rIL-13) treatment directly suppresses gluconeogenesis in hepatocytes, as evaluated by RNA-seq and validated by real-time quantitative PCR (RT–qPCR).

a, Experimental schematic for RNA-seq analysis of primary hepatocytes. Primary hepatocytes were isolated from C57BL/6 mice. Hepatocytes were treated with cAMP or glucagon for 3 h in the presence or absence of IL-13. **b**, Venn diagram showing 136 common differentially expressed genes (DEG) upregulated by cAMP and downregulated by IL-13 (upper panel). The table in the lower panel shows Kyoto Encyclopedia of Genes and Genomes (KEGG) pathways enriched in these DEGs. **c**, Heatmap showing the expression levels of genes and associated KEGG pathways, including *G6pc*, *Pck1*, and *Hnf4a*. **d**, RT–qPCR analysis of the *Hnf4a* downstream gluconeogenic enzymes *G6pc* and *Pck1* in IL-13-treated primary hepatocytes (normalized to *L32*) (n=3 per group). Unpaired one-sided Student's *t* test. $^{*}P < 0.05$; $^{**}P < 0.01$; $^{***}P < 0.001$. Each bar and its error bars represent the mean ± SD.

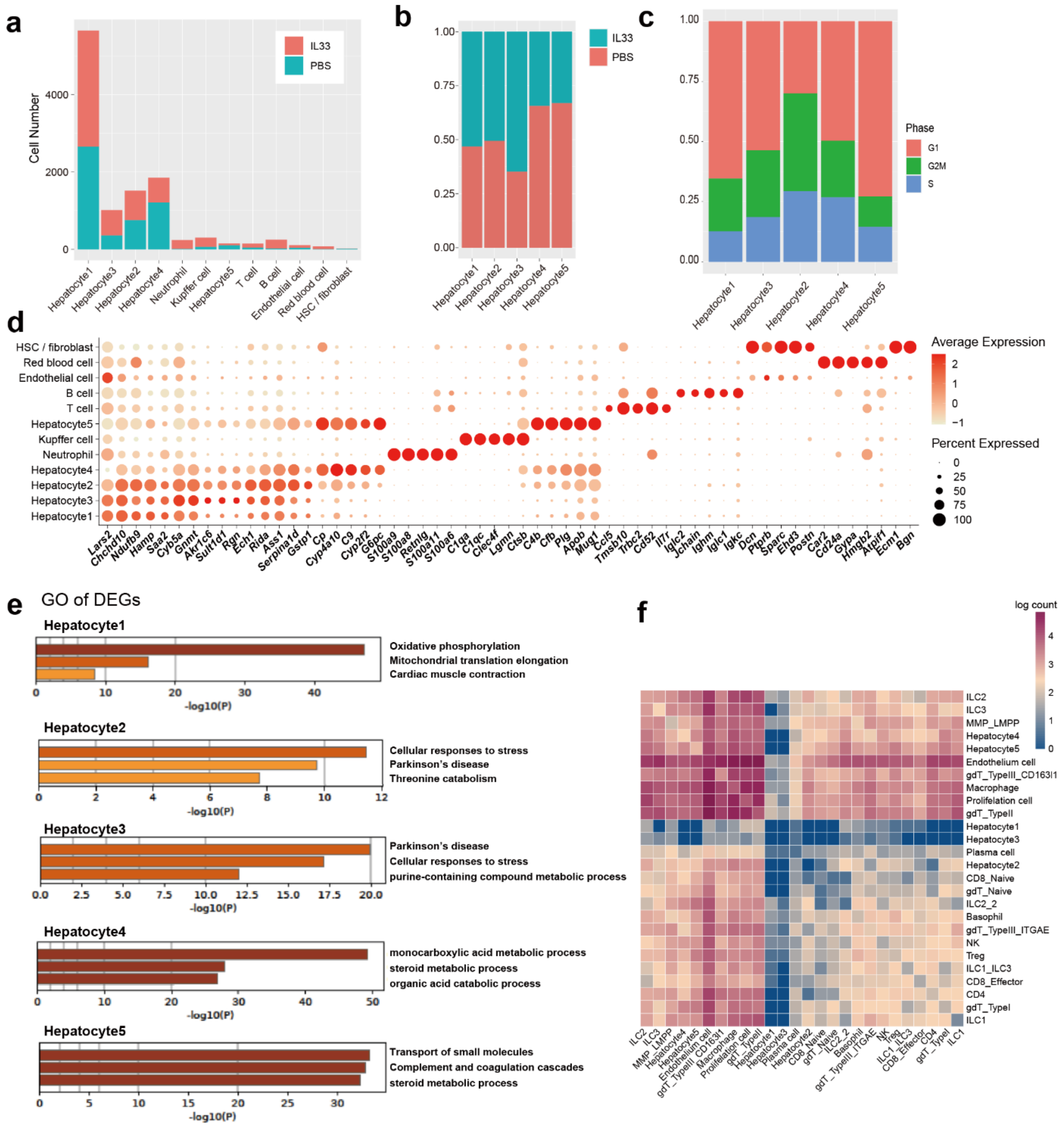

Supplementary Figure 5. | Characteristics of all cell clusters, including hepatocyte clusters. Single-cell RNA sequencing (scRNA-seq) data of hepatocyte-enriched cells in liver tissue (n = 11,327 single cells from the liver) from phosphate-buffered saline- and recombinant IL-33 (rIL-33)-treated livers. **a**, Cell numbers of the cell types within each treatment condition. **b,** Cell number ratio of each hepatocyte cluster within each treatment condition. **c,** Cell cycle phase within each hepatocyte cluster. **d,** Dot plot showing differentially expressed genes (DEG) for each cell type as defined by the FindAllMarkers function. **e**, Enriched Gene Ontology terms in each hepatocyte cluster (hepatocyte clusters 1−5). **f**, CellPhoneDB analysis. The interaction heatmap plots the total numbers of receptor and ligand interactions for the indicated cell types.

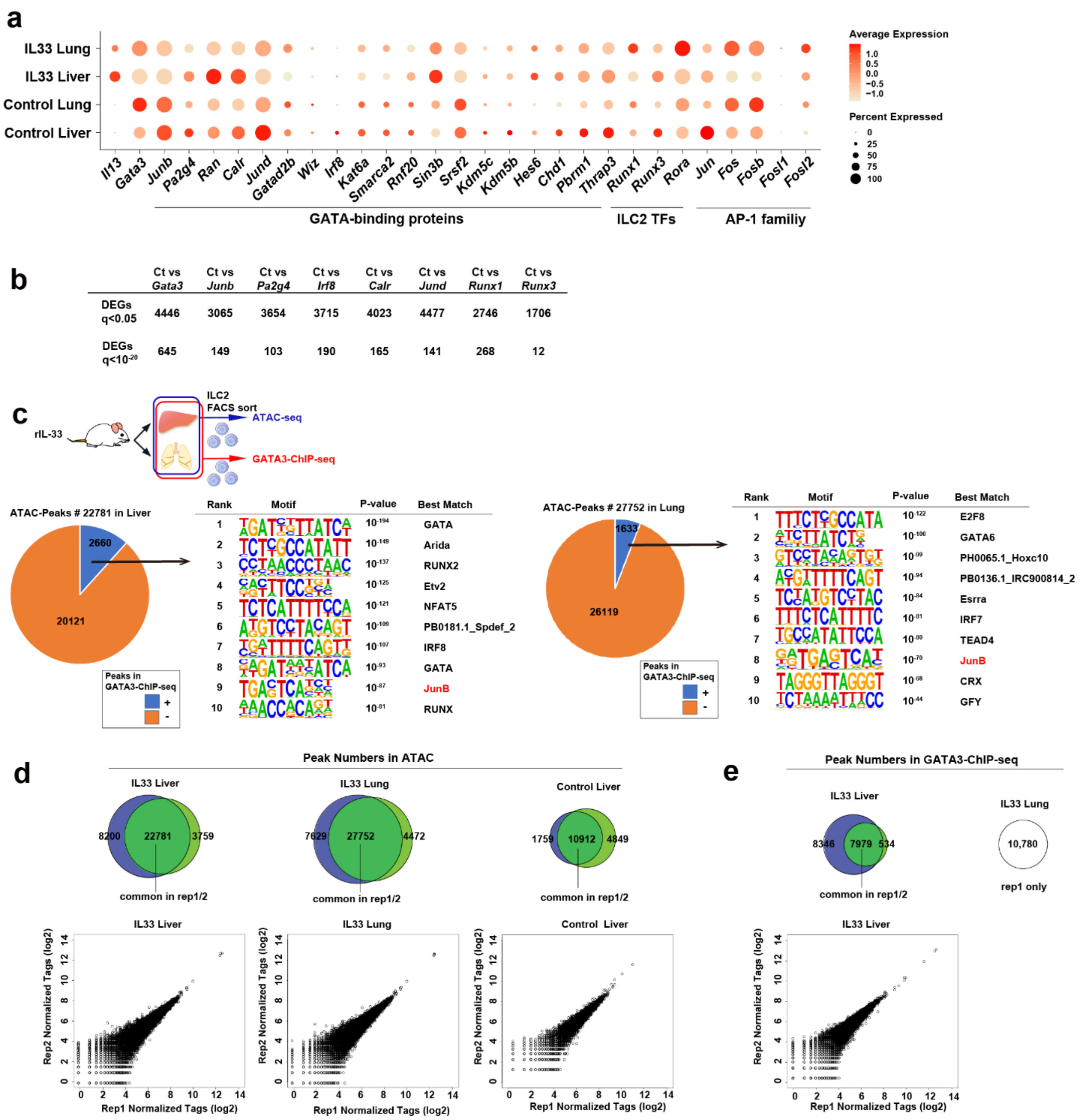

Supplementary Figure 6. | AP-1 family members bind to GATA3 in liver and lung group 2 innate lymphoid cells (ILC2) and suppress GATA3 function to induce *Il13* expression. a, Expression levels of *Il13* and GATA3-binding proteins, ILC2 transcription factors, and AP-1 family members, as determined by single-cell RNA sequencing (scRNA-seq) data. **b**, Number of differentially expressed genes (DEG) defined by the threshold q < 0.05 and q < 10^{-20}. Data for DEGs between ILC2s treated with Ct si and each siRNA are shown (n=3). **c**, Experimental schema of assay for transposase-accessible chromatin with high-throughput sequencing (ATAC-seq) and GATA3 chromatin immunoprecipitation sequencing (ChIP-seq). ILC2s were sorted from the livers and lungs of recombinant IL-33 (rIL-33)-treated mice. The pie chart shows the fraction of GATA3-bound ATAC-

seq peaks in the liver (left) and lungs (right). A motif analysis was conducted for the GATA3-bound ATAC-seq peaks, and the result is shown with a P value and corresponding DNA binding protein for each enriched motif. **d**, Venn diagram of ATAC-seq peaks from two biological replicates (upper). Scatter plot of the ATAC-seq normalized tags (log_2) between two replicates (lower). The results in rIL-33-treated liver (left), rIL-33-treated lung (middle), and phosphate-buffered saline (PBS)-treated liver (right) ILC2s are shown. **e**, Venn diagram of GATA3 ChIP-seq peaks from two biological replicates in rIL-33-treated liver ILC2s (upper left) and corresponding scatter plot of the ChIP-seq normalized tags (lower left). The number of GATA3 ChIP-seq peaks in rIL-33-treated lung ILC2s is also shown (upper right).

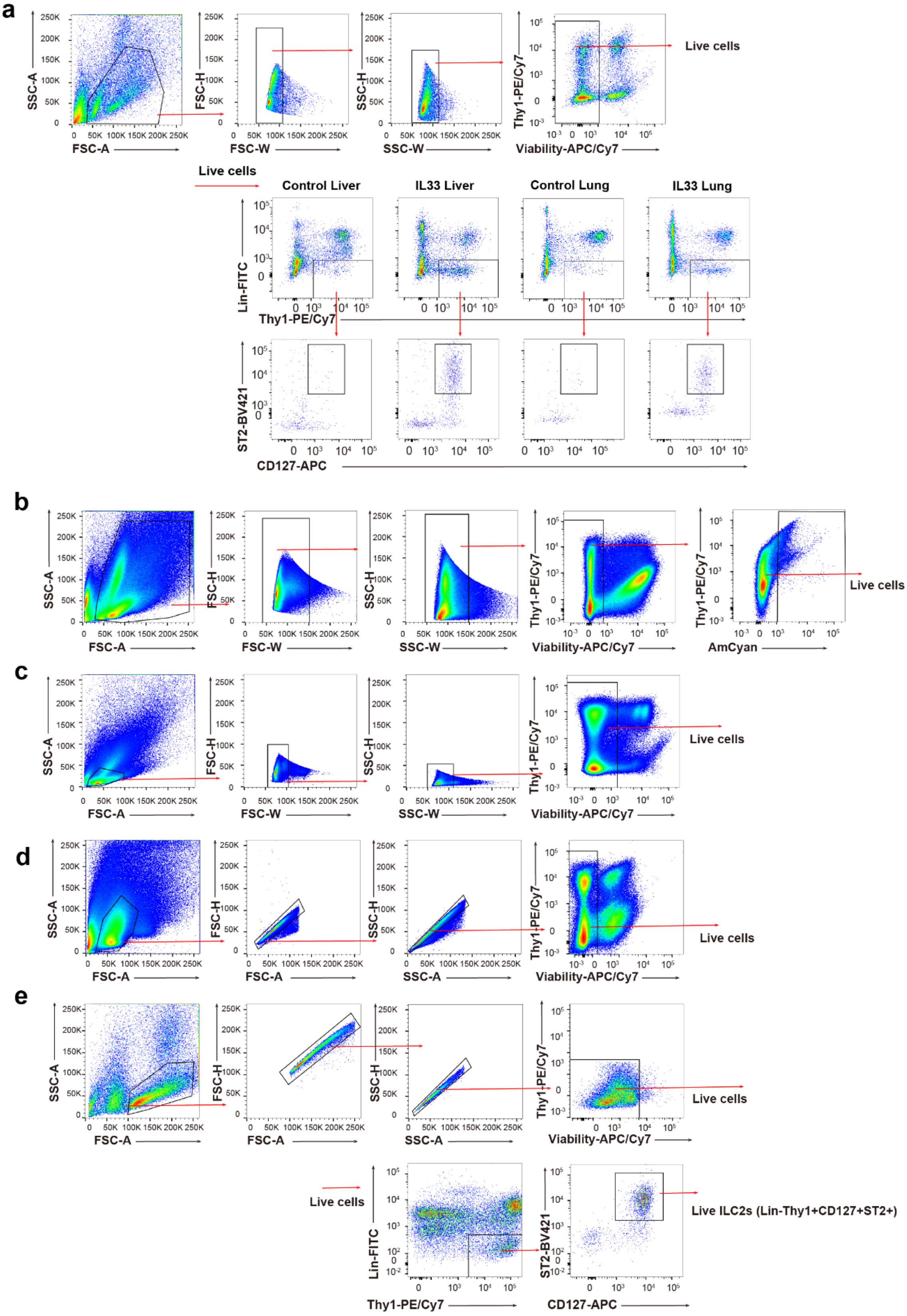

Supplementary Figure 7. | Gating strategies for cell sorting.

a, Gating strategy to sort ILC2s (Lin$^-$Thy1$^+$CD127$^+$ST2$^+$) from wild-type BALB/c mice treated with

Phosphate-buffered saline (PBS) or rIL-33 presented on Fig. 2a and 3a. **b**, Gating strategy to sort ILC2s ($Lin^-Thy1^+CD127^+ST2^+$) from wild-type BALB/c mice treated with Phosphate-buffered saline (PBS) or rIL-33 presented on Fig. 3h. **c**, Gating strategy to sort ILC2s ($Lin^-Thy1^+CD127^+ST2^+$) from 3-month normal diet (ND)-fed mice or HFD-fed BALB/c mice on Supplementary Fig. 2d. **d**, Gating strategy to sort ILC2s ($Lin^-Thy1^+CD127^+ST2^+$) from 3-month normal diet (ND)-fed mice or HFD-fed BALB/c mice on Supplementary Fig. 2e. **e**, Gating strategy to sort T cells ($CD3^+$) or ILC2s ($Lin^-Thy1^+CD127^+ST2^+$) from wild-type BALB/c mice on Supplementary Fig. 3b.

Supplementary Table 1

Primer sequences

List of primers for quantitative

(qPCR)

Target	Forward	Reverse
L32	GCTGCCATCTGTTTTACGG	TGACTGGTGCCTGATGAACT
Il4	CATCGGCATTTTGAACGAG	CGAGCTCACTCTCTGTGGTG
Il5	ACATTGACCGCCAAAAAGAG	ATCCAGGAACTGCCTCGTC
Il6	GCTACCAAACTGGATATAATCAGGA	CCAGGTAGCTATGGTACTCCAGAA
Il9	GCCTCTGTTTTGCTCTTCAGTT	GCATTTTGACGGTGGATCAT
Il13	CCTCTGACCCTTAAGGAGCTTAT	CGTTGCACAGGGGAGTCT
Gata3	TTATCAAGCCCAAGCGAAG	TGGTGGTGGTCTGACAGTTC
Il1rl1	GCTGAGGAATAAAGATGGCTAGG	GCTCTCTGAGGTAGGGTCCA
G6pc	TGTGTCTGGTAGGCAA	AACATCGGAGTGACCT
Pck1	ATCCCAACTCGAGATTCTGC	CCCAGGCAGGGTCAATAAT
Pcx	AATGTCCGGCGTCTGGAGTA	ACGCACGAAACACTCGGAT
Fbp1	CACCGCGATCAAAGCCATCT	CCAGTCACATTGGTTGAGCCA
Ppargc1a	TATGGAGTGACATAGAGTGTGCT	GTCGCTACACCACTTCAATCC
Pfkl	GGAGGCGAGAACATCAAGCC	GCACTGCCAATAATGGTGCC
Pklr	GAACATTGCACGACTCAACTTC	CAGTGCGTATCTCGGGACC
Hnf4a	AAGGTGCCAACCTCAATTCATC	CACATTGTCGGCTAAACCTGC
Areg	AAGAAAACGGGACTGTGCAT	GGCTTGGCAATGATTCAACT
Tnf	TCTTCTCATTCCTGCTTGTGG	GGTCTGGGCCATAGAACTGA
Junb	TCACGACGACTCTTACGCAG	CCTTGAGACCCCGATAGGGA
Pa2g4	GCAGGAGCAAACTATCGCC	ACCAAAGATCGAAGCACCCG
Irf8	AGACCATGTTCCGTATCCCCT	CACAGCGTAACCTCGTCTTCC
Calr	GCAGACCCTGCCATCTATTTC	TCGGACTTATGTTTGGATTCGAC
Jund	GAAACGCCCTTCTATGGCGA	CAGCGCGTCTTTCTTCAGC
Runx1	TGGTGGAGGTACTAGCTGACC	CGAGTAGTTTTCATCGTTGCCTG
Runx3	GACTCCTTCCCCAACTATACACC	CGCTGTTCTCGCCCATCTT
Jun	TTCCTCCAGTCCGAGAGCG	TGAGAAGGTCCGAGTTCTTGG
Fos	CGGGTTTCAACGCCGACTA	TGGCACTAGAGACGGACAGAT
Fosb	CCTCCGCCGAGTCTCAGTA	CCTGGCATGTCATAAGGGTCA
Fosl2	CACGCCGAGTCCTACTCCA	GTGGGCTGTACCATCCACTG

List of primers for chromatin immunoprecipitation with quantitative PCR (ChIP–qPCR)

**Target**	**Forward**	**Reverse**
STAT3 binding motif (*G6pc*)	GCTTGGTTGTGTGCTTTGCCTAGC	GCTGACCTTAAATTCTCTCTGTAGCC
STAT3 binding motif (*Pck1*)	GTTGCTCAAGTGCCAC	GTAGACCCTTCAGTGTC
STAT3 binding motif2 (*Hnf4a*)	GATGAGGACCAGATTTGCCGA	AAACTACCAGCCTGCCTTCTC
NC (*Hnf4a*)	AAGCTGGCCTCAAACTCACA	ATTCTGGCACTTGGAGGTGG
JUNB binding motif 1-1	CCCCTGGTCTCTGCTTTGTTG	TCCTTTAGCGGCCACTGGAT
JUNB binding motif 1-2	TCTGCTTTGTTGGGCATTATCTG	TGTCCCAGACCCTTCTCAAT
JUNB binding motif 2	GTGGCAGATCCCTTGGAGGT	CCTTCTGCTTGTCTTGAGGGG
NC1 (*Il13*)	CAGGCTCAAGGCATTTGTCG	TGGATGACAGTGACAACCTCC
NC2 (*Il13*)	GCCACCTCTAAGACCTACAGC	TAAGGAGACTTGGTGAGCATGG

**Target**	**Note**
STAT3 binding motif (*G6pc*)	5 kb upstream from *G6pc* TSS
STAT3 binding motif (*Pck1*)	1 kb upstream from *Pck1* TSS
STAT3 binding motif2 (*Hnf4a*)	1.5 kb upstream from *Hnf4a* TSS
NC (*Hnf4a*)	5.3 kb upstream from *Hnf4a* TSS without STAT3 motif
JUNB binding motif 1-1	40 base upstream from *Il13* TSS
JUNB binding motif 1-2	40 base upstream from *Il13* TSS
JUNB binding motif 2	2.9 kb upstream from *Il13* TSS
NC1 (*Il13*)	11.1 kb upstream from *Il13* TSS without JUNB motif
NC2 (*Il13*)	11.5 kb upstream from *Il13* TSS without JUNB motif

Supplementary Table 2

List of genes ignored in the clustering of scRNA-seq samples.

"mt-“,"mmu","sno","Gm","Rn6","RNA","7SK","SNORD","SNORA","SCARNA","B3g", "Vmn","Mir","Rik","Snora","Snord","LOC","Rn4","OTTMUSG","Scarna","Rnu","Rmr","Rpl","Rps","AA","AB","AC","AF","AI","AL","AU","AV","AW","AY","Malat","Tpt1","B2m","Actb","Vim", "Tmsb4x","Eef","Fau","BC","B9d","BX","ERCC","Hist","Igkv","Olfr","RP","n-","BB","BY","B4g","CK","CN","CR","CT","Hbb","Hba","n-“.

Supplementary Table 3

The list of antibodies.

Surface marker antibodies for flowcytometry:

viability dye APC-Cy7 (eBioscience, 65-0865-14, 1:1000)

anti-CD16/CD32 (eBioscience, 14-0161-82, 93, 1:33)

anti-lineage cocktail-FITC

(BioLegend, 133302, 145-2C11; RB6-8C5; RA3-6B2; Ter-119; M1/70, 1:40)

anti-TCRβ-FITC (BioLegend, 109206, H57-597, 1:200)

anti-CD90.2-PE/Cy7 (BioLegend, 105326, 30-H12, 1:200)

anti-CD127-APC (BioLegend, 135012, A7R34, 1:100)

anti-ST2-BV421 (BioLegend, 145309, DIH9, 1:100)

anti-CD3E-FITC (BioLegend, 100203, 17A2, 1:200)

anti-TCRg-PE (BioLegend, 118107, GL3, 1:100)

Intracellular cytokine antibodies for flowcytometry:

anti-IL-13 (BioLegend, 159403, W17010B, 1:100)

Antibodies for immunoprecipitation:

anti-GATA3 (Santa Cruz, sc-268, 1 μg/ml ; R&D Systems, MAB26051, 1 μg/ml)

anti-JunB (Santa Cruz, sc-8051, 1:50; Cell signaling, C37F9 (#3753), 1:50)

anti-STAT3 (Cell signaling, 124H6 (#9139), 1:100; D3Z2G (#12640), 1:50)

anti-Flag M2 agarose (Sigma–Aldrich, A2220, 50 μl/sample)

Antibodies for western blot

anti-JunB (Sant Cruz, sc8051, 1 μg/ml)

anti-Flag (Sigma, A2220, 1 μg/ml)

anti-Tubulin α (Sigma, T6119, 1 μg/ml)

Antibodies for Immunofluorescence:

anti-KLRG1 (1:25, FITC conjugated, Biolegend, 138409)

rabbit anti-PCK1 (1:50, abcam, ab70358)

anti-CD3e (1:25, biotin conjugated, eBioscience, 13-0033-82)

Alexa Fluor 647 goat anti-rabbit antibody (1:50, Molecular Probes, A-21244)

Alexa Fluor 594 streptavidin (1:50, Jackson ImmmunoResearch, 016-580-084)
