# Supplementary material for: Liver group 2 innate lymphoid cells regulate blood glucose levels through IL-13 signaling and suppression of gluconeogenesis
Source: Nat Commun. 2022 Sep 15;13:5408. doi: 10.1038/s41467-022-33171-6 (PMC9478157; doi:10.1038/s41467-022-33171-6)
Supplement: Supplementary file 3 — Reporting Summary [file 41467_2022_33171_MOESM3_ESM.pdf]

nature portfolio

Corresponding author(s): Tomoaki Tanaka

Last updated by author(s): Aug 25, 2022

Reporting Summary

Nature Portfolio wishes to improve the reproducibility of the work that we publish. This form provides structure for consistency and transparency in reporting. For further information on Nature Portfolio policies, see our Editorial Policies and the Editorial Policy Checklist.

Statistics

For all statistical analyses, confirm that the following items are present in the figure legend, table legend, main text, or Methods section.

n/a	Confirmed	
☐	☒	The exact sample size (*n*) for each experimental group/condition, given as a discrete number and unit of measurement
☐	☒	A statement on whether measurements were taken from distinct samples or whether the same sample was measured repeatedly
☐	☒	The statistical test(s) used AND whether they are one- or two-sided<br>*Only common tests should be described solely by name; describe more complex techniques in the Methods section.*
☒	☐	A description of all covariates tested
☐	☒	A description of any assumptions or corrections, such as tests of normality and adjustment for multiple comparisons
☐	☒	A full description of the statistical parameters including central tendency (e.g. means) or other basic estimates (e.g. regression coefficient) AND variation (e.g. standard deviation) or associated estimates of uncertainty (e.g. confidence intervals)
☐	☒	For null hypothesis testing, the test statistic (e.g. *F*, *t*, *r*) with confidence intervals, effect sizes, degrees of freedom and *P* value noted<br>*Give P values as exact values whenever suitable.*
☒	☐	For Bayesian analysis, information on the choice of priors and Markov chain Monte Carlo settings
☒	☐	For hierarchical and complex designs, identification of the appropriate level for tests and full reporting of outcomes
☒	☐	Estimates of effect sizes (e.g. Cohen's *d*, Pearson's *r*), indicating how they were calculated

Our web collection on statistics for biologists contains articles on many of the points above.

Software and code

Policy information about availability of computer code

Data collection	FACS CantoII(BD), FACSymphony A3 (BD)and FACSAriaIII(BD) were used for flow cytometry data collection.<br>Sequenceing acquisition: Novaseq, HiSeq1500, NextSeq 500. ABI StepONEPlus was used for quantitative real-time PCR.
Data analysis	Flowjo 10.4.2<br>Prism (GraphPad) v9<br>Bowtie (v1.2.2)<br>Bowtie2 (v2.1.0)<br>HOMER (v4.10)<br>IGV (v2.9.4)<br>macs2 (v2.2.7.1)<br>R v3.6.1 or 4.0.4<br>TopHat v1.3.2<br>Cufflinks v2.0.2<br>edgeR v3.28.1<br>cellranger v4.0.0 or v6.0.0<br>Seurat v.3.1.2 or v4.0.4<br>monocle3 v0.2.3.3<br>CellPhoneDB v2.0.0<br>Mascot v2.5.1

For manuscripts utilizing custom algorithms or software that are central to the research but not yet described in published literature, software must be made available to editors and reviewers. We strongly encourage code deposition in a community repository (e.g. GitHub). See the Nature Portfolio guidelines for submitting code & software for further information.

Data

Policy information about availability of data

All manuscripts must include a data availability statement. This statement should provide the following information, where applicable:

- Accession codes, unique identifiers, or web links for publicly available datasets
- A description of any restrictions on data availability
- For clinical datasets or third party data, please ensure that the statement adheres to our policy

Bulk RNA-seq, ATAC-seq, ChIP-seq and single cell RNA-seq dataset will be deposited in the Gene Expression Omnibus (GEO) with the accession number GSE189287. The remaining data that support the findings of this study are available from the corresponding authors upon request. Source data are also provided as Source Data.

Field-specific reporting

Please select the one below that is the best fit for your research. If you are not sure, read the appropriate sections before making your selection.

☒ Life sciences ☐ Behavioural & social sciences ☐ Ecological, evolutionary & environmental sciences

For a reference copy of the document with all sections, see nature.com/documents/nr-reporting-summary-flat.pdf

Life sciences study design

All studies must disclose on these points even when the disclosure is negative.

Sample size	Sample sizes of experiments were chosen based on our previous research and similar reports on ILC2s or reports in which evaluating the glucose metabolisms (L. Ying et al, Nature Communications. 12, Article number: 7256 (2021); RR. Ricardo-Gonzalez et al, Nature Immunology. 19, 1093 (2018)). GraphPad PRISM software was used for statistical analyses. Unpaired t test were used to determine the statistical significance (p<0.05, one-tailed). We observed many statistically significant effects in the data, indicating that the effective sample size was sufficient for studying the phenomena of interest.
Data exclusions	No data were excluded from the analysis.
Replication	The experimental findings were reliably reproduced with at least two independent experiments in Fig. 1a-g, i, j; Fig. 3h, i; Fig. 4a-e, g, i; Fig. 5f, l, m; Fig. 6d; Fig. 7e, f, i; Fig. 8b, 8c; Supplementary Fig. 1a-d; Supplementary Fig. 2a-2h; Supplementary Fig. 3b; Supplementary Fig. 4d. The micrograph of immunofluorescence in Fig. 5f is representative of 24 locations of 5 independent mice. Western blotting in Fig. 7f is representative figures of two independent experiments. We performed scRNA-seq of ILC2s using pooled tissues of 2-6 mice in each group (Fig. 3). ATAC-seq and ChIP-seq (Fig. 7c, d, Supplementary Fig. 6c, d, e) were performed with duplicates only except for GATA3-ChIP-seq from IL-33 treated lung (Supplementary Fig. 6c right, 6e). RNA-seq (Fig. 7j) of cultured ILC2s were performed with triplicates.
Randomization	Age- and sex-matched mice were used in experiments to control the covariates.
Blinding	Although main investigator were not blinded to group allocation, all procedure of sampling and data collection (including blood glucose measuring, flowcytometry, RT-qPCR, ELISA, hepatocyte isolation) are performed not by single investigator, but some independent investigators. We also utilized many omics approach (Bulk-RNA-seq, ATAC-seq, ChIP-seq, single cell-RNAseq, MS/MS analysis, Metabolome) in this study, which enables us to interpret data without bias.

Reporting for specific materials, systems and methods

We require information from authors about some types of materials, experimental systems and methods used in many studies. Here, indicate whether each material, system or method listed is relevant to your study. If you are not sure if a list item applies to your research, read the appropriate section before selecting a response.

Materials & experimental systems

n/a	Involved in the study
☐	☒ Antibodies
☐	☒ Eukaryotic cell lines
☒	☐ Palaeontology and archaeology
☐	☒ Animals and other organisms
☒	☐ Human research participants
☒	☐ Clinical data
☒	☐ Dual use research of concern

Methods

n/a	Involved in the study
☐	☒ ChIP-seq
☐	☒ Flow cytometry
☒	☐ MRI-based neuroimaging

Antibodies

Antibodies used	Surface marker antibodies for flowcytometry:<br>viability dye APC-Cy7 (eBioscience, 65-0865-14, 1:1000)<br>anti-CD16/CD32 (eBioscience, 14-0161-82, 93, 1:33)<br>anti-lineage cocktail-FITC<br>(BioLegend, 133302, 145-2C11; RB6-8C5; RA3-6B2; Ter-119; M1/70, 1:40)<br>anti-TCRβ-FITC (BioLegend, 109206, H57-597, 1:200)<br>anti-CD90.2-PE/Cy7 (BioLegend, 105326, 30-H12, 1:200)<br>anti-CD127-APC (BioLegend, 135012, A7R34, 1:100)<br>anti-ST2-BV421 (BioLegend, 145309, DIH9, 1:100)<br>anti-CD3E-FITC (BioLegend, 100203, 17A2, 1:200)<br>anti-TCRg-PE (BioLegend, 118107, GL3, 1:100)<br><br>Intracellular cytokine antibodies for flowcytometry:<br>anti-IL-13 (BioLegend, 159403, W17010B, 1:100)<br><br>Antibodies for immunoprecipitation:<br>anti-GATA3 (Santa Cruz, sc-268, 1 µg/ml ; R&D Systems, MAB26051, 1 µg/ml)<br>anti-JunB (Santa Cruz, sc-8051, 1:50; Cell signaling, C37F9 (#3753), 1:50)<br>anti-STAT3 (Cell signaling, 124H6 (#9139), 1:100; D3Z2G (#12640), 1:50)<br>anti-Flag M2 agarose (Sigma–Aldrich, A2220, 50 µl/sample)<br><br>Antibodies for western blot<br>anti-JunB (Sant Cruz, sc8051, 1 µg/ml)<br>anti-Flag (Sigma, A2220, 1 µg/ml)<br>anti-Tubulin [illegible] (Sigma, T6119, 1 µg/ml)<br><br>Antibodies for Immunofluorescence:<br>anti-KLRG1 (1:25, FITC conjugated, Biolegend, 138409)<br>rabbit anti-PCK1 (1:50, abcam, ab70358)<br>anti-CD3e (1:25, biotin conjugated, eBioscience, 13-0033-82)<br>Alexa Fluor 647 goat anti-rabbit antibody (1:50, Molecular Probes, A-21244)<br>Alexa Fluor 594 streptavidin (1:50, Jackson ImmmunoResearch, 016-580-084)
Validation	All the antibodies were validated by manufacturer, and the information about the validation can be found on the manufacturer's website through the links below:<br>https://www.fishersci.com/us/en/home.html<br>https://www.biolegend.com<br>https://www.scbt.com/home<br>https://www.cellsignal.com/?country=USA Abcam<br>https://www.jacksonimmuno.com/

Eukaryotic cell lines

Policy information about cell lines

Cell line source(s)	The ILC2 cell line ILC2/b6 generously gifted from Dr. Q Yang
Authentication	Functionally in repeated tests; by cell surface phenotype and expression profiles including GATA3 downstream genes.
Mycoplasma contamination	ILC2/b6 tested negative for mycoplasma contamination.
Commonly misidentified lines<br>(See ICLAC register)	No commonly misidentified cell lines were used.

Animals and other organisms

Policy information about studies involving animals; ARRIVE guidelines recommended for reporting animal research

Laboratory animals	BALB/c, nude (Foxnnu/nu), C57BL/6, NSG, NOD/Scid/Il2Rγnull (NSG), Il13-/- males of 6-8 weeks were used.<br>All mice were allowed ad libitum access to food and water and were maintained in specific pathogen-free conditions in a 22°C temperature-controlled room with a 12 h light-12 h dark cycle. Humidity maintained in the range of 40-70%.
Wild animals	This study did not involve wild animals.
Field-collected samples	This study did not involve samples collected from the field.
Ethics oversight	The research proposals were reviewed by the ethics committee for animals at Chiba University.

Note that full information on the approval of the study protocol must also be provided in the manuscript.

ChIP-seq

Data deposition

☒ Confirm that both raw and final processed data have been deposited in a public database such as GEO.

☒ Confirm that you have deposited or provided access to graph files (e.g. BED files) for the called peaks.

Data access links<br>*May remain private before publication.*	We are preparing for deposit raw and processed data listed below in GSE189287 [https://www.ncbi.nlm.nih.gov/geo/query/acc.cgi?acc=GSE189287].
Files in database submission	___ATAC-seq___<br>LiverILC2_1.fastq.gz<br>LiverILC2_2.fastq.gz<br>33LiverILC2_1.fastq.gz<br>33LiverILC2_2.fastq.gz<br>33LungILC2_1.fastq.gz<br>33LungILC2_2.fastq.gz<br><br>LiverILC2_1_mm10_tag.ucsc.bedGraph.gz<br>LiverILC2_2_mm10_tag.ucsc.bedGraph.gz<br>33LiverILC2_1_mm10_tag.ucsc.bedGraph.gz<br>33LiverILC2_2_mm10_tag.ucsc.bedGraph.gz<br>33LungILC2_1_mm10_tag.ucsc.bedGraph.gz<br>33LungILC2_2_mm10_tag.ucsc.bedGraph.gz<br><br>___GATA3-ChIP-seq___<br>LiverILC2_rep1.fastq.gz<br>LiverILC2_rep2.fastq.gz<br>LungILC2_rep1.fastq.gz<br><br>LiverILC2.rep1.mm10_tag.ucsc.bedGraph.gz<br>LiverILC2.rep2.mm10_tag.ucsc.bedGraph.gz<br>LungILC2.rep1.mm10_tag.ucsc.bedGraph.gz<br><br>___bulkRNA-seq_Hepatocyte___<br>cAMP1.fastq.gz<br>cAMP2.fastq.gz<br>cAMP3.fastq.gz<br>cAMPrIL13_1.fastq.gz<br>cAMPrIL13_2.fastq.gz<br>cAMPrIL13_3.fastq.gz<br>Gluca1.fastq.gz<br>Gluca2.fastq.gz<br>Gluca3.fastq.gz<br>GlucarIL13_1.fastq.gz<br>GlucarIL13_2.fastq.gz<br>GlucarIL13_3.fastq.gz<br>rest1.fastq.gz<br>rest2.fastq.gz<br>rest3.fastq.gz<br><br>cAMP1.bedgraph.gz<br>cAMP2.bedgraph.gz<br>cAMP3.bedgraph.gz<br>cAMPrIL13_1.bedgraph.gz<br>cAMPrIL13_2.bedgraph.gz<br>cAMPrIL13_3.bedgraph.gz<br>Gluca1.bedgraph.gz<br>Gluca2.bedgraph.gz<br>Gluca3.bedgraph.gz<br>GlucarIL13_1.bedgraph.gz<br>GlucarIL13_2.bedgraph.gz<br>GlucarIL13_3.bedgraph.gz<br>rest1.bedgraph.gz<br>rest2.bedgraph.gz<br>rest3.bedgraph.gz<br><br>FPKMCuffDiff.xlsx<br>TagCountCuffDiff.xlsx<br><br>___scRNA-seq_ILC2___<br>33LiverILC2_S1_L004_R1_001.fastq.gz

33LiverILC2_S1_L004_R2_001.fastq.gz
33LungILC2_S2_L004_R1_001.fastq.gz
33LungILC2_S2_L004_R2_001.fastq.gz
CtLiverILC2_S3_L004_R1_001.fastq.gz
CtLiverILC2_S3_L004_R2_001.fastq.gz
CtLungILC2_S4_L004_R1_001.fastq.gz
CtLungILC2_S4_L004_R2_001.fastq.gz
FilteredFiles/33LiverILC2
FilteredFiles/33LungILC2
FilteredFiles/CtLiverILC2
FilteredFiles/CtLungILC2
(each of 4 directory above include barcodes.tsv.gz, features.tsv.gz and matrix.mtx.gz)
SupplementaryTable1_AllMarkers_1.csv
SupplementaryTable2_ILC2subclusterMarkers.csv

___scRNA-seq_Hepatocyte___
HepatocyteIL33_S1_L001_R1_001.fastq.gz
HepatocyteIL33_S1_L001_R2_001.fastq.gz
HepatocytePBS_S1_L001_R1_001.fastq.gz
HepatocytePBS_S1_L001_R2_001.fastq.gz
FilteredFiles/Hepato33
FilteredFiles/HepatoCt
(each of 2 directory above include barcodes.tsv.gz, features.tsv.gz and matrix.mtx.gz)
SupplementaryTable3_AllMarkers_2.csv
SupplementaryTable4_HepatocyteMarkers.csv

Genome browser session
(e.g. UCSC)

ChIP-seq data were mapped to the mouse genome build mm10 using Bowtie2 (v2.1.0)
with default settings.
ATAC-seq data were mapped to the mouse genome build mm10 using Bowtie2 (v2.1.0)
with --broad option.
For visualization in the IGV, HOMER makeTagDirectory and makeUCSCfile commands were used.

Methodology

Replicates

Data analysis is based on reproducible ChIP-seq peaks in two replicate samples except IL33-treated Lung sample.

Sequencing depth

ChIP-seq libraries were sequenced on Illumina Hiseq1500 in single read mode with the read length 50 bp. Fastq files were produced for GATA3-ChIP-seq (7.4-17.2 million reads per sample) and ATAC-seq (14.2-24.7 million reads per sample).

Antibodies

anti-GATA3 (Santa Cruz, sc-268; R&D Systems, MAB26051)

Peak calling parameters

ChIP-seq: macs2 callpeak -f SAM -g mm -n. ATAC-seq: macs2 callpeak --broad -f SAM -g mm -n.

Data quality

Peak reproducibility was determined by HOMER mergePeaks command.

Software

macs2 (v2.2.7.1)
Bedtools (v.2.17.0)
Bowtie2 (v2.1.0)
HOMER (v4.10)

Flow Cytometry

Plots

Confirm that:

☒ The axis labels state the marker and fluorochrome used (e.g. CD4-FITC).

☒ The axis scales are clearly visible. Include numbers along axes only for bottom left plot of group (a 'group' is an analysis of identical markers).

☒ All plots are contour plots with outliers or pseudocolor plots.

☒ A numerical value for number of cells or percentage (with statistics) is provided.

Methodology

Sample preparation

The isolation of ILC2s from the liver or lung:

Male wild-type , Il13+/- and Il13-/- BALB/c, NSG mice were euthanized and perfused with PBS, then liver and lung ILC2s were harvested. Mouse livers and lungs were digested using a gentle MACS Octo Dissociator with a Heaters (Miltenyi) using a liver dissociation kit (Miltenyi, 130-105-807, program: 37C_m_LIDK_1) and a lung dissociation kit (Miltenyi, 130-095-927, m_lung_01, incubation at 37°C for 30 min, and then m_lung_02 program), respectively. After filtration through a 70-µm cell strainer, the cells were collected by centrifugation at 500g for 5 min at 4°C and resuspended in 40% Percoll Plus (GE

	Healthcare). Then, 75% Percoll was gently added below the 40% Percoll layer. After centrifugation at 700g for 20 min at room temperature (RT), the cells at the interface were collected in PBS containing 1% FBS and 2 mM EDTA.
Instrument	gentleMACS™ Octo Dissociator with Heaters were used for cell sampling.<br>FACS CantoII(BD) and Symphony A3(BD) were used for multi-parameter analysis.<br>Cell sorting was performed on FACSAriaIII(BD).
Software	FlowJo(v10.4.2)
Cell population abundance	Before sorting experiment the cytometer was calibrated using CS&T and Accudrop beads as described by the manufacturer (BD Biosciences). ILC2s defined as Lin− (CD3e−Ly6G−Ly6c−CD11b−CD45R−TER119−TCRb−) CD127+Thy1+ST2+ were sort purified by FACS ARIA III.
Gating strategy	Doublets were excluded using forward light-scatter gating followed by gating on lymphocytes based on FSC/SSC. Dead cells were excluded by gating on Viability dye negative cells. ILC2s were defined as live, Lin− (CD3e−Ly6G−Ly6c−CD11b−CD45R −TER119−TCRb−) CD127+Thy1+ST2+ cells, indicated in Supplementary Figures.

☒ Tick this box to confirm that a figure exemplifying the gating strategy is provided in the Supplementary Information.
