# Supplementary figures and images for: Liver group 2 innate lymphoid cells regulate blood glucose levels through IL-13 signaling and suppression of gluconeogenesis
Source: Nat Commun. 2022 Sep 15;13:5408. doi: 10.1038/s41467-022-33171-6 (PMC9478157; doi:10.1038/s41467-022-33171-6)

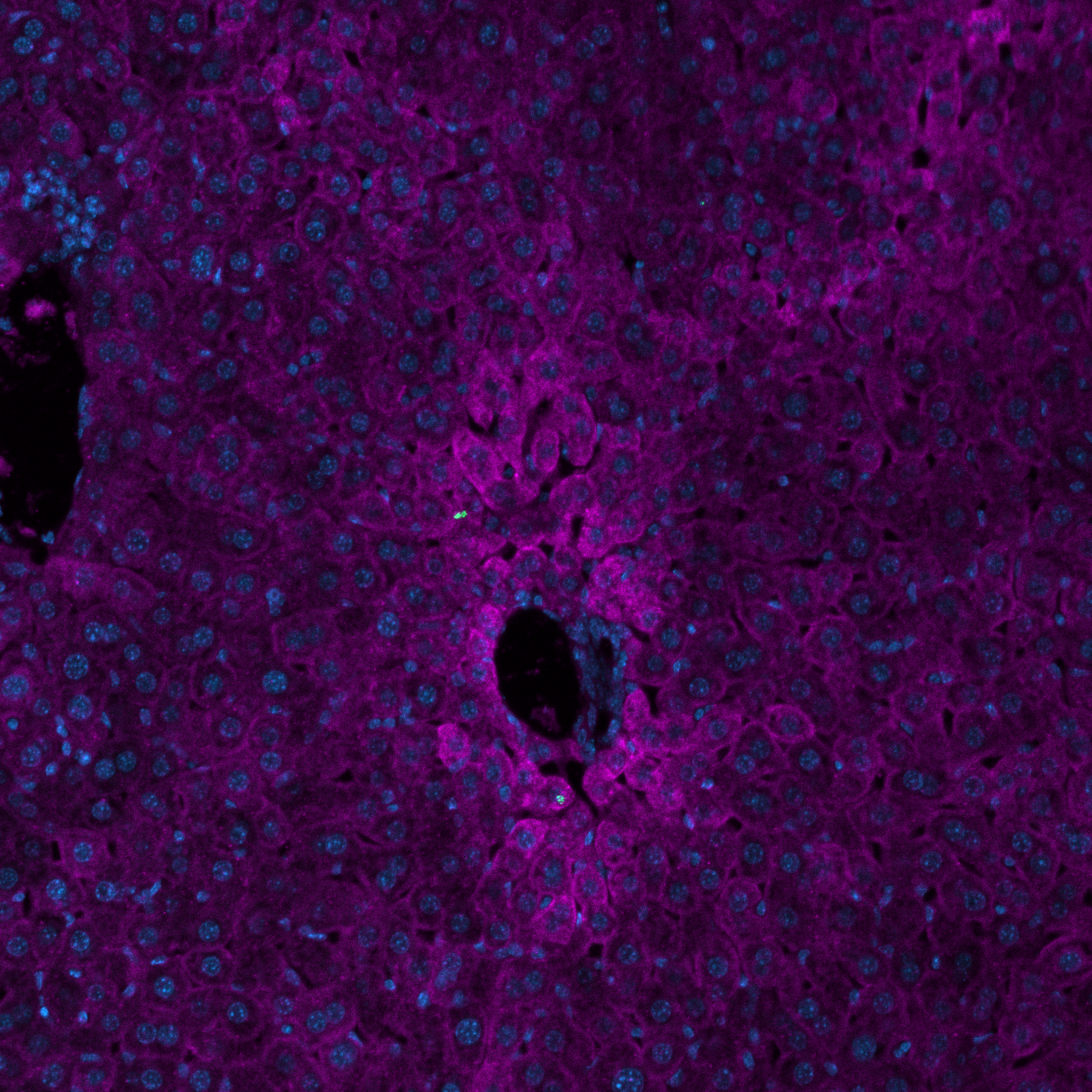

Supplement: Supplementary file 4 — Source Data [file 41467_2022_33171_MOESM4_ESM.zip › source.data.folder.zipped.20220820/Fig.4f PCK1-647_KLRG1-FITC_CD3e-594 x20.tif]

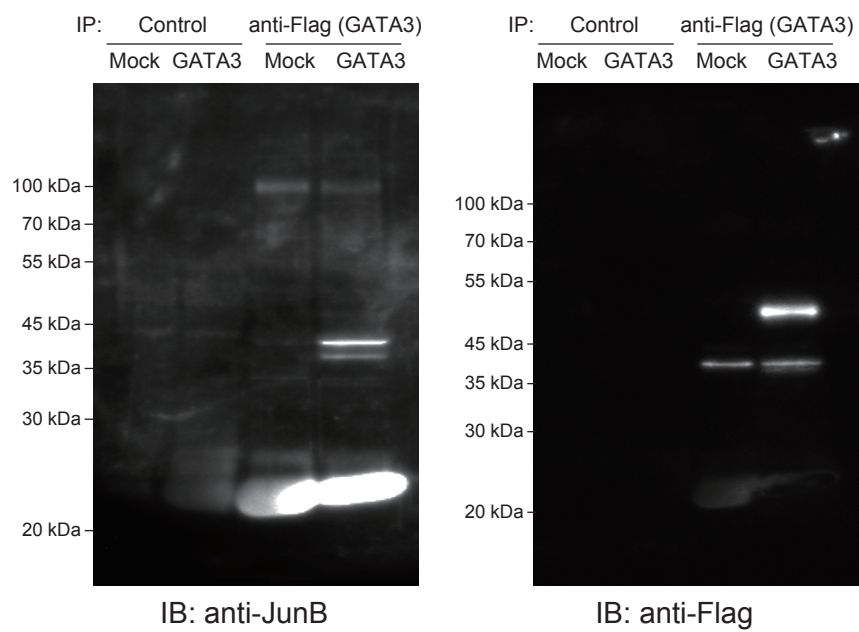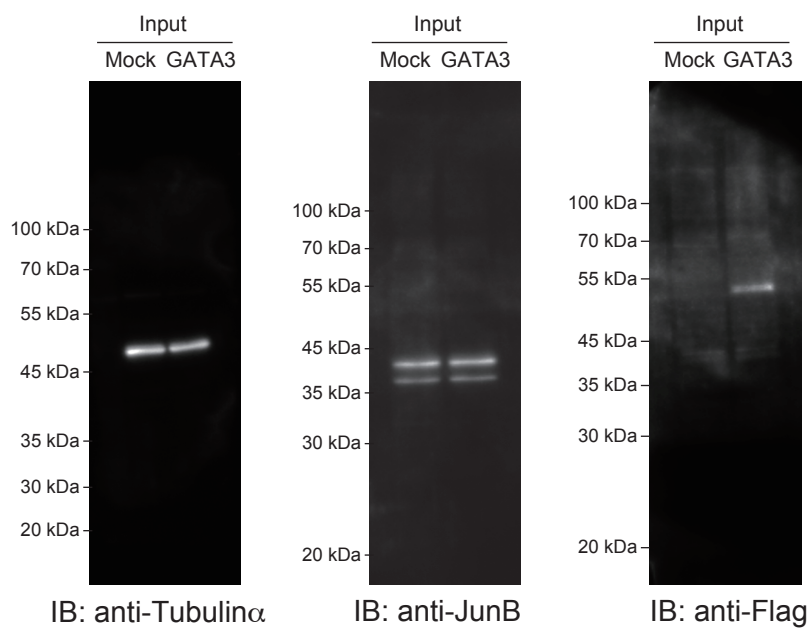

Supplement: Supplementary file 4 — Source Data [file 41467_2022_33171_MOESM4_ESM.zip › source.data.folder.zipped.20220820/Fig.5f ILC2 GATA3-JunB.uncropped.versions.of.gels.pdf]
